# Molecular detection of *Helicobacter* spp. and *Fusobacterium gastrosuis* in pigs and wild boars and its association with gastric histopathological alterations

**DOI:** 10.1186/s13567-022-01101-5

**Published:** 2022-10-08

**Authors:** Francisco Cortez Nunes, Teresa Letra Mateus, Emily Taillieu, Sílvia Teixeira, Nuno Carolino, Alexandra Rema, Sofie De Bruyckere, Fátima Gärtner, Freddy Haesebrouck, Irina Amorim

**Affiliations:** 1 School of Medicine and Biomedical Sciences (ICBAS), University of Porto, 4050-313 Porto, Portugal; 2 Institute for Research and Innovation in Health (i3S), University of Porto, 4200-135 Porto, Portugal; 3 Institute of Molecular Pathology and Immunology of the University of Porto (IPATIMUP), 4200-135 Porto, Portugal; 4 CISAS-Centre for Research and Development in Agrifood Systems and Sustainability, Escola Superior Agrária, Instituto Politécnico de Viana do Castelo, 4900-347 Viana do Castelo, Portugal; 5 Laboratory for Integrative and Translational Research in Population Health (ITR), EpiUnit, Instituto de Saúde Pública da Universidade do Porto, Rua das Taipas, no. 135, 4050-091 Porto, Portugal; 6 Associate Laboratory for Animal and Veterinary Sciences (AL4AnimalS) Quinta de Prados, Veterinary and Animal Research Centre (CECAV), UTAD, 5000-801 Vila Real, Portugal; 7 Department of Veterinary Sciences, Vasco da Gama Research Center (CIVG), Vasco da Gama University School, Coimbra, Portugal; 8 Centre for Interdisciplinary Research in Animal Health (CIISA), University of Lisboa, Lisbon, Portugal; 9 National Institute for Agrarian and Veterinarian Research, I.P., Vale de Santarém, Portugal; 10 Department of Pathobiology, Pharmacology and Zoological Medicine, Faculty of Veterinary Medicine, Ghent University, Ghent, Belgium

**Keywords:** *Sus scrofa*, stomach, gastritis, gastric pathology, One Health

## Abstract

Besides *Helicobacter pylori*, a Gram-negative bacterium that may cause gastric disorders in humans, non-*Helicobacter pylori* helicobacters (NHPH) may also colonize the stomach of humans and animals. In pigs, *H. suis* can induce gastritis and may play a role in gastric ulcer disease, possibly in association with *Fusobacterium gastrosuis*. In the present study, gastric samples from 71 slaughtered pigs and 14 hunted free range wild boars were tested for the presence of DNA of *F. gastrosuis* and gastric *Helicobacter* species associated with pigs, dogs cats and humans, using species-specific PCR assays, followed by sequencing of the amplicon. These gastric samples were also histopathologically evaluated. Almost all the pigs presented gastritis (95.8%). *Helicobacter* spp*.* were detected in 78.9% and *F. gastrosuis* in 35.2% of the animals. *H. suis* was the most frequently identified *Helicobacter* species (57.7% of the animals), followed by a *H. pylori*-like species (50.7%) and less often *H. salomonis* and *H. felis* (each in 2.8% of the animals). *H. suis* was most often detected in the glandular (distal) part of the stomach (*pars oesophagea* 9.9%, oxyntic mucosa 35.2%, antral mucosa 40.8%), while the *H. pylori*-like species was mainly found in the non-glandular (proximal) part of the stomach (*pars oesophagea* 39.4%, oxyntic mucosa 14.1%, antral mucosa 4.2%). The great majority of wild boars were also affected with gastritis (71.4%) and *Helicobacter* spp*.* and *F. gastrosuis* were detected in 64.3% and 42.9% of the animals, respectively. *H. bizzozeronii* and *H. salomonis* were the most frequently detected *Helicobacter* species, while a *H. pylori-*like species and *H. suis* were only occasionally identified. These findings suggest that these microorganisms can colonize the stomach of both porcine species and may be associated with gastric pathology. This should, however, be confirmed through bacterial isolation. This is the first description of the presence of *F. gastrosuis* DNA in the stomach of wild boars and a *H. pylori-*like species in the *pars oesophagea* of the porcine stomach.

**Supplementary Information:**

The online version contains supplementary material available at 10.1186/s13567-022-01101-5.

## Introduction

*Helicobacter* species are Gram-negative, spiral-shaped motile bacteria that colonize the gastrointestinal tract of humans and a wide range of animals [1–4]. Studies have been done over the years to investigate their association with gastrointestinal and extra-gastrointestinal diseases [5]. In humans, *Helicobacter pylori* (*H. pylori*) is the most common gastric pathogen, with an estimated prevalence of 44.3% worldwide [6]. Other spiral-shaped non-*Helicobacter pylori* helicobacters (NHPH) have also been associated with gastric diseases in humans. These gastric helicobacters can be responsible for development of gastritis, gastroduodenal ulcers, gastric adenocarcinoma and mucosa-associated lymphoid tissue (MALT) lymphoma [2, 7, 8].

Zoonotic NHPH such as *H. suis*, *H. felis*, *H. bizzozeronii*, *H. salomonis*, and *H. heilmannii*, with an estimated prevalence ranging from 0.2 to 6% in symptomatic humans, naturally colonize the stomach of dogs, cats and pigs [6, 9].

*Helicobacter suis* is found mainly in the fundic and pyloric gland zone of the pig stomach [10]. This bacterium presents tropism for the gastric acid-producing parietal cells [11]. Its prevalence appears to be very low prior to weaning, but increases rapidly thereafter, being very high at slaughter age (77%) and in adults (>90%) [10–12]. *H. suis* infection causes gastritis, decreased daily weight gain, and may play a role in induction of gastric ulcers possibly in association with *Fusobacterium gastrosuis* (*F. gastrosuis*), further affecting animal production and welfare [13–15].

Fusobacteria are Gram-negative, non-sporing, strict anaerobic bacilli. They tend to form long filamentous rods, described as fusiform, spindle-shaped short with rounded ends [16]. There are 27 recognized species, including *F. gastrosuis* [17]. Fusobacteria can be involved in a wide range of mixed infections. Depending on the host species they can play different roles: in humans they can be associated with gingivitis, dental plaque formation, dental and periodontal abscesses and liver abscesses; while in pigs, they have been associated with lameness, facial skin necrosis and gastric ulceration [17].

*Fusobacterium gastrosuis* was primarily identified in the *pars oesophagea* of the pig stomach [17]. This anatomical region may be affected by ulceration, with a prevalence of up to 93% [18]. Gastric ulceration can lead to discomfort, decrease in daily weight gain, and sudden dead [17]. It is known that gastric ulceration is multifactorial and multiple agents can be involved such as *H. suis* and *F. gastrosuis* [17].

Several *Helicobacter* spp. have been described to have a zoonotic potential and therefore close contact between humans, domestic animals, and wild animals deserves more attention [19–23]. Although reservoirs of wild and domestic animals can be considered important sources of emerging infectious diseases, it is the human impact on ecological systems that determines the level of risk at the human/animal interface upon the occurrence of emerging zoonotic diseases [22, 23]. From an eco-epidemiological perspective, wild boars have an important role in spreading several pathogens [24–26].

The aim of this study is to determine the presence of gastric helicobacters and *F. gastrosuis* in a group of pigs and wild boars and describe the most relevant gastric histopathological alterations associated.

## Materials and methods

### Sample collection

Pig gastric tissues samples were collected from a total of 71 domestic pigs slaughtered at two slaughterhouses in the center of Portugal: one in Coimbra (*n* = 46, from 3 different herds) and another in Santarém (*n* = 25, from 2 different herds). All sampled animals were older than 6 months according to the slaughter animal registries.

Wild boar gastric tissue samples were collected from 14 hunted animals during two national campaigns, one in the north and other in the center of Portugal (Vila Real and Coimbra districts, respectively). Based on tooth evaluation [26], all sampled animals were older than 9 months.

From each animal, two samples were collected from the three different anatomical gastric regions: *pars oesophagea*, *antrum* and *fundus*, using a sterile disposable Kruuse® biopsy punch of 8 mm per site. After collection, from each region one sample was fixed in 10% phosphate-buffered formalin for histopathology and the other sample was stored at −20 °C for further DNA extraction and molecular analysis.

Gastric tissue sampling was performed within one hour and 4 h after slaughter, in pigs and wild boars respectively. The animals were not slaughtered, euthanized, or hunted to carry out this study, and the fresh gastric tissue specimens were purchased and obtained as sub-products derived from the normal meat inspection activity occurring in the slaughterhouses or during the national campaigns. Additionally, any of these actions were performed solely for research purposes and the researchers had no influence in the selection and execution of the slaughters nor in the national hunting campaign’s inspection procedures. Ethical approval was obtained from the i3S Animal Welfare and Ethics Review Body (ref. 2021-4).

### Sample evaluation

#### Histological examination

After fixation, gastric tissues were routinely processed, paraffin-embedded and stained with hematoxylin and eosin (HE) for histopathology. The severity of gastritis was scored according to the human Updated Sydney System [14, 27] classification, with some modifications [13]. The same criteria were applied to the *pars oesophagea*, as described by Yamasaki et al., although this gastric region is not included within the human Updated Sydney System [28, 29]. Diffuse infiltration with inflammatory cells and the presence of lymphoid aggregates and lymphoid follicles in the mucosa and submucosa were also evaluated [13, 14, 27, 28] (Table 1).

**Table 1:** **Histological parameters used to establish a scoring system based on the Updated Sydney System and De Witte et al. and Gastritis overall scoring system** [13]

Parameter		Scoring
Inflammatory cells (lymphocytes, plasm cells, neutrophils, eosinophils)	Absent	0
	Mild infiltration (<5 cells per HPF)	1
	Moderate infiltration (≥5–20 cells per HPF)	2
	Severe infiltration (≥20 cells per HPF)	3
Lymphoid follicles in the superficial and deep mucosa	No aggregates of lymphoid cells	0
	Presence of one aggregate of lymphoid cells with minimal organization into a follicular structure	1
	Presence of at least one large follicle measuring at least 300 µm in diameter and/or more than one small aggregate of lymphoid cells per histological section	2
	At least two large lymphoid follicles measuring at least 300 µm in diameter per histological section and/or deformation of the mucosal caused by large lymphoid follicles	3
Gastritis overall scoring system		
Score		
0	Normal	
1	Mild gastritis	
2	Moderate gastritis	
≥ 3	Severe gastritis	

HPF: high power field, i.e., 400× total magnification.

A semi quantitative estimation of the overall inflammatory degree for each gastric area (*pars oesophagea*, oxyntic and antral mucosa) was calculated using a composite score which consisted of adding the partial values previously obtained [13, 27]. Additionally, each gastric section was also microscopically evaluated for the presence of hyperplasia, erosion, ulceration, and fibrosis.

Finally, the overall inflammatory score was correlated with the presence of *H. pylori*, *H. suis*, *H. felis*, *H. salomonis*, *H. bizzozeronii*, *H. heilmannii*, *H. ailurogastricus* and *F. gastrosuis* using Pearson Correlation coefficient.

#### DNA extraction, PCR conditions, and sequencing

DNA was extracted from the frozen tissue samples, using EXTRACTME® DNA tissue Kit (BLIRT, Poland) according to the manufacturer’s instructions.

All the samples were tested for the presence of *H. pylori*, *H. suis*, *H. salomonis*, *H. bizzozeronii*, *H. felis*, *H. heilmannii*, *H. ailurogastricus* and *F. gastrosuis* DNA through conventional PCR, according to previously described protocols (Additional file 1) [2, 18, 30, 31]. As can be seen in Additional file 1: Table S1, for detection of *H. pylori* DNA, two different PCR tests were used, one targeting the *ureAB* gene and the other one targeting the *glmM* gene.

Aliquots of each PCR product were electrophoresed on 1.5% agarose gel stained with Xpert Green Safe DNA gel stain (GRISP, Porto, Portugal) and examined for the presence of specific fragment under UV light. DNA fragment size was compared with the standard molecular weight, 100 bp DNA ladder (GRISP, Porto, Portugal) and the molecular weight of the positive controls (Additional file 1). For negative control, distilled water was used.

To exclude false-positive samples, the amplicons from each positive sample were sequenced. Bidirectional sequencing was performed using Sanger method at the genomics core facility of the Institute of Molecular Pathology and Immunology of the University of Porto. Sequence editing and multiple alignments were performed using MegaX Molecular Evolutionary Genetic Analysis version 10.1.8. The sequences obtained were subject to the basic local alignment search tool (BLAST) using the non-redundant nucleotide database.

### Statistical analysis

Statistical analysis was performed using SAS®9.4 (SAS Institute Inc., 2019. Copyright® 2019 SAS Institute Inc., Cary, NC, USA). Correlations between different variables (erosion, ulceration, hyperplasia, fibrosis and presence of *Helicobacter* spp. and or *F. gastrosuis*) were investigated using Pearson correlation coefficient, *r* results of 0 meaning no correlation, *r* < 0.3 low degree of correlation, *r* ≥ 0.3 to *r* < 0.5 moderate degree of correlation, and *r* ≥ 0.5 to 1 high degree of correlation. Differences were considered statistically significant at *p* ≤ 0.05.

Differences between the different variables (erosion, ulceration, hyperplasia, fibrosis and presence of *Helicobacter* spp. and or *F. gastrosuis*) of the *pars oesophagea*, oxyntic mucosa, and antral mucosa were investigated using non-parametric Kruskal–Wallis, Chi-Square test. A *p*-value ≤ 0.05 was considered to be significant.

## Results

### Pigs

A total of 426 gastric samples were collected from 71 animals: 213 for histopathology and 213 for molecular evaluation.

#### Histopathology

Only 192 of the 213 available samples for histopathology were microscopically evaluated and classified, since 21 samples were in poor preservation conditions impairing histological examination. Thus 57 samples from *pars oesophagea*, 69 from the oxyntic mucosa, and 66 from the antral mucosa were analyzed.

**Figure 1:**
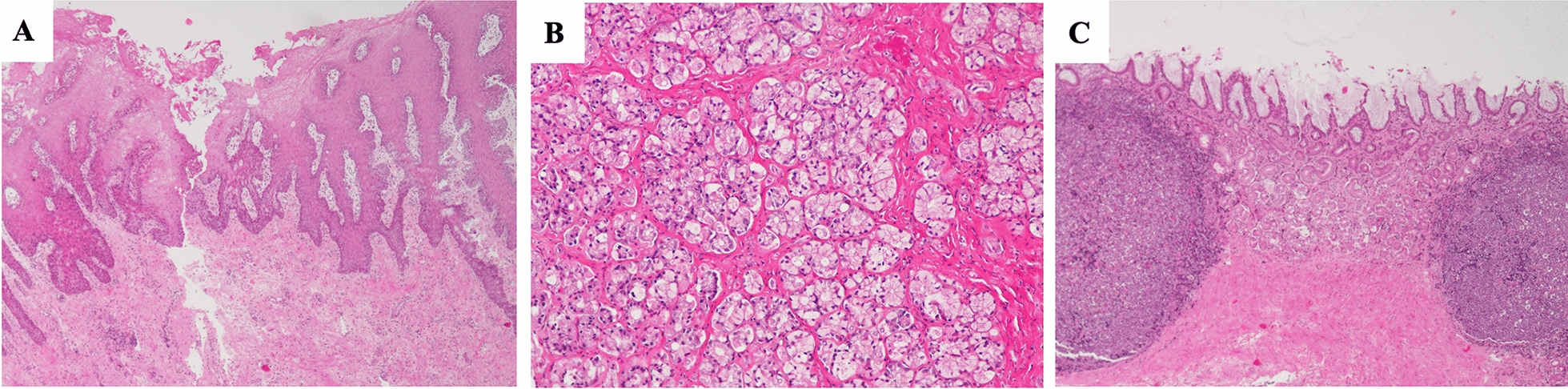
**Main histopathological features observed in the different regions of pigs’ stomach.** **A** Erosion and irregular and papillary hyperplasia of the lining epithelium of *pars oesophagea.* HE, ×40; **B** Mild inflammation of oxyntic mucosa. HE, ×100. **C** Severe inflammation and deformation of the antral mucosal caused by large lymphoid follicles and fibrosis. HE, ×40.

Normal histological features were only observed in 3/71 (4.2%) pigs. Signs of inflammation were diagnosed in 95.8% of pigs. Indeed, 52 out of 57 samples (91.2%) of *pars oesophagea*, 60 out of 69 samples (86.9%) of oxyntic mucosa and all the samples of antral mucosa analyzed (100%) exhibited gastritis (Figure 1). Among samples with gastritis, hyperplasia was present in 28/52 (53.8%) of *pars oesophagea* and fibrosis in 49/60 (81.6%) of oxyntic and in 44/66 (66.6%) of antral mucosa (Table 2).

**Table 2:** **Results of the histopathology of the different gastric regions of pigs’ stomach**

	Pars oesophagea		Oxyntic mucosa		Antral mucosa	
Number of samples per gastric zone	*N* = 57		*N* = 69		*N* = 66	
	*n*/*N*	%	*n*/*N*	%	*n*/*N*	%
Normal mucosa	5/57	8.8	9/69	13.0	0/66	0.0
Score 0						
Fibrosis	0/5	0.0	6/9	66.7	0/0	0.0
Erosion	1/5	20.0	0/9	0.0	0/0	0.0
Ulceration	0/5	0.0	0/9	0.0	0/0	0.0
Hyperplasia	3/5	60.0	0/9	0.0	0/0	0.0
Mild gastritis	16/57	28.1	28/69	40.6	2/66	3.0
Score 1						
Fibrosis	2/16	12.5	23/28	82.1	1/2	50.0
Erosion	4/16	25.0	1/28	3.6	1/2	50.0
Ulceration	2/16	12.5	0/28	0.0	0/2	0.0
Hyperplasia	8/16	50.0	0/28	0.0	0/2	0.0
Moderate gastritis	9/57	15.8	13/69	18.8	3/66	4.5
Score 2						
Fibrosis	0/9	0.0	12/13	92.3	3/3	100.0
Erosion	0/9	0.0	2/13	15.4	0/3	0.0
Ulceration	0/9	0.0	0/13	0.0	0/3	0.0
Hyperplasia	6/9	66.7	0/13	0.0	0/3	0.0
Severe gastritis	27/57	47.4	19/69	27.5	61/66	92.4
Score ≥ 3						
Fibrosis	0/27	0.0	14/19	73.7	40/61	65.6
Erosion	5/27	18.5	1/19	5.3	12/61	19.7
Ulceration	1/27	3.7	0/19	0.0	21/61	34.4
Hyperplasia	14/27	51.9	0/19	0	2/61	3.3

Remarkably, almost all samples of antral mucosa presented severe inflammation (61/66 or 92.4%) and amongst these, fibrosis was also a common finding (40/61 or 65.6%) (Table 2). When comparing the three different gastric zones, there were statistically significant differences for the presence of erosion, ulceration, hyperplasia, and fibrosis (*p* ≤ 0.05) (Additional file 2).

#### Presence of *Helicobacter* spp. and *F. gastrosuis* DNA through PCR analysis

In ten pigs, no DNA amplification was achieved, neither for *Helicobacter* spp*.* nor for *F. gastrosuis*.

Out of all animals (*n* = 71), *Helicobacter* spp*.* were detected in 56 stomachs (78.9%) with *H. suis* being the most frequently identified species (41/71 or 57.7%), followed by a *H. pylori*-like species (36/71 or 50.7%) and less often *H. salomonis* and *H. felis* (2/71 or 2.8% each).

Regarding the *H. pylori* PCR results, 36/71 samples amplified *H. pylori* DNA with homologies ranging from 96.3 to 100% (Additional file 3) using the *ureAB* gene primers but no amplification was achieved when using the *glmM* gene primers. Therefore, these cases were reclassified as *H. pylori*-like.

Positive results for *F. gastrosuis* were obtained in 25 out of the 71 pigs (35.2%), although *F. gastrosuis* was mainly found in association with helicobacters (20/71 or 28.2%) rather than alone (5/71 or 7.0%). Indeed, in 27/71 of the positive samples (38.0%), only one species was identified, while mixed infections with two or more distinct bacteria were detected in 34/71 (47.9%) (Table 3). The most frequent bacterial combination was *H. pylori*-like + *H. suis* (12/71 or 16.9%) and *H. pylori*-like + *H. suis* + *F. gastrosuis* (10/71 or 14.1%) (Table 3).

**Table 3:** ***Helicobacter*** **spp. and** ***F. gastrosuis*** **DNA PCR positive samples per pigs’ stomach**

Specific PCR-positive results	*n*/*N*	%
Overall identification		
*Helicobacter* spp*.* and/or *F. gastrosuis*	61/71	85.9
*Helicobacter* spp*.*	56/71	78.9
*H. suis*	41/71	57.7
*H. pylori*-like	36/71	50.7
*H. felis*	2/71	2.8
*H. salomonis*	2/71	2.8
*F. gastrosuis*	25/71	35.3
Single bacteria		
*H. suis*	14/71	19.7
*H. pylori*-like	7/71	9.9
*F. gastrosuis*	5/71	7.0
*H. felis*	1/71	1.4
Multiple bacteria		
*H. pylori*-like + *H. suis*	12/71	16.9
*H. pylori*-like + *F. gastrosuis*	5/71	7.0
*H. suis* + *F. gastrosuis*	4/71	5.6
*H. pylori*-like + *H. salomonis*	1/71	1.4
*H. suis* + *H. felis*	1/71	1.4
*H. pylori*-like + *H. suis* + *H. salomonis* + *F. gastrosuis*	1/71	1.4
*H. pylori*-like + *H. suis* + *F. gastrosuis*	10/71	14.1

Bacteria were differently distributed throughout the porcine gastric compartments (Table 4). *H. pylori*-like DNA was mostly detected in the *pars oesophagea* (28/71, 39.4%), while *H. suis* was most frequently identified in the oxyntic (25/71, 35.2%) and antral mucosa (29/71, 40.8%). There was also a statistically highly significant moderate degree of correlation regarding the presence of *H. pylori*-like + *H. suis* DNA in the *pars oesophagea,* as well as a statistically significant low degree of correlation between the presence of *H. pylori*-like and *H. suis* in the *pars oesophagea* (*r* = 0.29 *p* ≤ 0.05) and oxyntic mucosa (*r* = 0.25, *p* ≤ 0.05) (Table 4).

**Table 4:** ***Helicobacter*** **spp. and** ***F. gastrosuis*** **detected through PCR in the different regions of pigs’ stomach**

	Pars oesophagea		*r*	*p*	Oxyntic mucosa		*r*	*p*	Antral mucosa		*r*	*p*
Number of samples	*N* = 71				*N* = 71				*N* = 71			
	*n*/*N*	%			*n*/*N*	%			*n*/*N*	%		
Single bacteria												
*H. pylori*-like	28/71	39.4			10/71	14.1			3/71	4.2		
*H. suis*	7/71	9.9			25/71	35.2			29/71	40.8		
*H. felis*	0/71	0.0			2/71	2.8			0/71	0.0		
*H. salomonis*	0/71	0.0			0/71	0.0			2/71	2.8		
*F. gastrosuis*	12/71	16.9			4/71	5.6			10/71	7.6		
Multiple bacteria												
*F. gastrosuis* + *H. pylori*-like	8/71	11.3	0.29	0.03	1/71	1.4	0.25	0.04	1/71	1.4	0.11	0.38
*H. pylori*-like + *H. suis*	6/71	8.5	0.36	< 0.01	1/71	1.4	−0.07	0.56	2/71	2.8	−0.05	0.71
*F. gastrosuis* + *H. suis*	0/71	0.0	−0.04	0.79	2/71	2.8	0.20	0.09	4/71	5.6	−0.03	0.79
*H. suis* + *H. felis*	0/71	0.0	–	–	1/71	1.4	0.05	0.69	0/71	0.0	–	–
*F. gastrosuis* + *H. pylori*-like + *H. suis*	1/71	1.4	–	–	1/71	1.4	–	–	0/71	0.0	–	–

*r*: Pearson correlation coefficient.

p: *p*-value ≤ 0.05 was considered to be significant.

Regarding the correlation between the presence of both bacteria and the respective histological findings, a statistically highly significant moderate correlation was observed between the presence of *H. felis* and erosion in the oxyntic mucosa (*r* = 0.33, *p* ≤ 0.01); a statistically significant low degree of correlation between the presence of *H. pylori-*like and erosion (*r* = 0.23, *p* ≤ 0.05) in the antral mucosa as well as a statistically significant correlation between the presence of *F. gastrosuis* and ulceration of the antral mucosa (*r* = 0.31, *p* ≤ 0.05) (Table 5).

**Table 5:** **Correlation between the PCR-positive results obtained in the different stomach regions and the gastric histological findings in pigs**

		Erosion			Ulceration			Hyperplasia			Fibrosis		
	n/N	10/57	*r*	*p*	3/57	*r*	*p*	31/57	*r*	*p*	2/57	*r*	*p*
Pars oesophagea													
*H. pylori*-like	28/57	1/10	−0.11	0.40	1/3	−0.03	0.81	14/31	0.04	0.64	2/2	0.20	0.13
*H. suis*	7/57	1/10	−0.03	0.83	0	−0.08	0.57	4/31	0.04	0.77	0	−0.07	0.63
*H. felis*	0/57	0	–	–	0	–	–	0	–	–	0	–	–
*F. gastrosuis*	12/57	2/10	−0.04	0.74	0	−0.12	0.39	7/31	−0.01	0.93	0	–0.10	0.46

	n/N	4/69	*r*	*p*	0	*r*	*p*	0	*r*	*p*	4	*r*	*p*
Oxyntic mucosa													
*H. pylori*-like	10/69	0	−0.10	0.40	0	–	–	0	–	–	0	−0.21	0.08
*H. suis*	25/69	2/4	0.07	0.56	0	–	–	0	–	–	2/4	0.0	0.16
*H. felis*	2/69	1/4	0.33	< 0.01	0	–	–	0	–	–	1/4	0.15	0.19
*F. gastrosuis*	4/69	1/4	0.20	0.09	0	–	–	0	–	–	1/4	0.08	0.51

	n/N	13/66	*r*	*p*	21/66	*r*	*p*	2/66	*r*	*p*	44/66	*r*	*p*
Antral mucosa													
*H. suis*	29/66	5/13	−0.02	0.85	10/21	0.13	0.31	1/2	−0.04	0.78	16/44	0.02	0.86
*H. pylori*-like	3/66	2/13	0.23	0.02	0	−0.01	0.31	0	−0.04	0.77	1/44	−0.02	0.86
*H. felis*	0/66	0	–	–	0	–	–	0	–	–	0	–	–
*F. gastrosuis*	10/66	2/13	−0.11	0.36	4/21	0.31	0.011	1/2	0.08	0.50	3/44	−0.20	0.11

*r*: Pearson correlation coefficient.

*p*: *p*-value ≤ 0.05 was considered to be significant.

### Wild boars

A total of 84 samples were collected from 14 animals: 42 samples for histopathological examination and 42 samples for molecular evaluation.

#### Histopathology

Only 30 of the 42 available samples for histopathology were microscopically evaluated and classified, since 12 were in poor preservation conditions impairing histological examination (10 of the *pars oesophagea*, 10 of the oxyntic mucosa and 10 of the antral mucosa).

**Figure 2:**
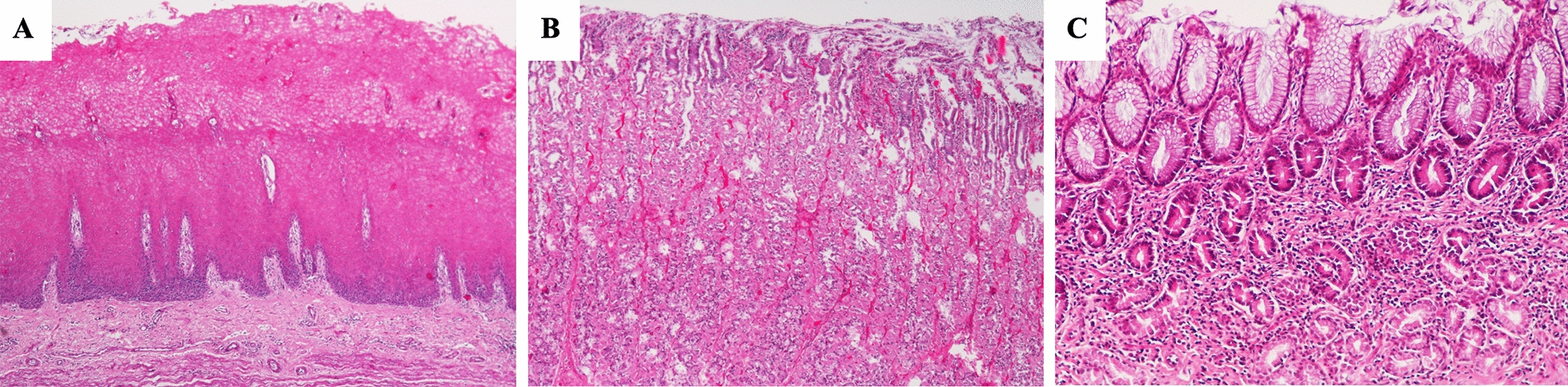
**Main histopathological features observed in the different regions of wild boars’ stomach.** **A** Hyperkeratosis and hyperplasia of the lining epithelium of *pars oesophagea.* HE, ×40; **B** Mild inflammation of oxyntic mucosa. HE, ×40 and **C** Moderate and diffuse inflammatory infiltration of antral mucosa. HE, ×100.

Normal histological features were not identified in wild boars. Microscopically, gastritis was diagnosed in 8 out of 10 samples of *pars oesophagea* (80.0%), 4 out of 10 samples of oxyntic mucosa (40.0%), and 9 out of 10 samples of antral mucosa (90.0%). In most representative cases of *pars oesophagea* the inflammation was mild (4/10 or 40.0%), whereas in the antral mucosa the inflammation was severe (7/10 or 70.0%) (Figure 2).

Regarding the cases diagnosed with gastritis of *pars oesophagea* (8/10), 3 out of 8 were associated with erosion (37.5%) and 2 out of 8 with hyperplasia (25.0%) (Table 6).

**Table 6:** **Results of the histopathology of the different gastric regions of wild boars’ stomach**

	Pars oesophagea		Oxyntic mucosa		Antral mucosa	
Number of samples per gastric zone	*N* = 10		*N* = 10		*N* = 10	
	*n*/*N*	%	*n*/*N*	%	*n*/*N*	%
Normal mucosa	2/10	20.0	6/10	60.0	1/10	10.0
Score 0						
Fibrosis	0/2	0.0	0/6	0.0	0/1	0.0
Erosion	1/2	50.0	0/6	0.0	0/1	0.0
Ulceration	0/2	0.0	0/6	0.0	0/1	0.0
Hyperplasia	0/2	0.0	0/6	0.0	0/1	0.0
Mild gastritis	4/10	40.0	1/10	10.0	1/10	10.0
Score 1						
Fibrosis	0/4	0.0	0/1	0.0	0/1	0.0
Erosion	2/4	50.0	0/1	0.0	0/1	0.0
Ulceration	0/4	0.0	0/1	0.0	0/1	0.0
Hyperplasia	1/4	25.0	0/1	0.0	0/1	0.0
Moderate gastritis	2/10	20.0	2/10	20.0	1/10	10.0
Score 2						
Fibrosis	0/2	0.0	0/2	0.0	0/1	0.0
Erosion	0/2	0.0	0/2	0.0	0/1	0.0
Ulceration	0/2	0.0	0/2	0.0	0/1	0.0
Hyperplasia	0/2	0.0	1/2	50.0	0/1	0.0
Severe gastritis	2/10	20.0	1/10	10.0	7/10	70.0
Score ≥ 3						
Fibrosis	0/2	0.0	0/1	0.0	0/7	0.0
Erosion	1/2	25.0	0/1	0.0	0/7	0.0
Ulceration	0/2	0.0	0/1	0.0	0/7	0.0
Hyperplasia	1/2	25.0	0/1	0.0	0/7	0.0

#### *Helicobacter* spp. and *F. gastrosuis* identification through PCR analysis

In 3 out of 14 wild boars, no DNA amplification was achieved, neither for *Helicobacter* spp*.* nor for *F. gastrosuis*. Out of all animals, *Helicobacter* spp*.* were detected in 9 sampled stomachs (78.6%) with both *H. bizzozeronii* and *H. salomonis* being the most frequently identified species (4/11 or 36.4%), while a *H. pylori*-like species (2/11 or 18.2%) and *H. suis* (1/11 or 9.1%) were less often detected (Table 7).

**Table 7:** ***Helicobacter*** **spp. and** ***F. gastrosuis*** **DNA PCR positive samples per wild boars’ stomach**

Specific PCR-positive results	*n*/*N*	%
Overall identification		
*Helicobacter* spp*.* + *F. gastrosuis*	11/14	78.6
*Helicobacter* spp.	9/14	64.3
*H. bizzozeronii*	4/14	28.6
*H. salomonis*	4/14	28.6
*H. pylori*-like	2/14	14.3
*H. suis*	1/14	7.1
*F. gastrosuis*	6/14	42.9
Single bacteria		
*F. gastrosuis*	2/14	14.3
*H. salomonis*	2/14	14.3
*H. pylori*-like	0/14	0.0
*H. suis*	1/14	7.1
*H. bizzozeronii*	1/14	7.1
Multiple bacteria		
*H. bizzozeronii* + *F. gastrosuis*	2/14	14.3
*H. pylori* + *H. salomonis*	1/14	7.1
*H. pylori*-like + *F. gastrosuis*	1/14	7.1
*H. bizzozeronii* + *H. salomonis* + *F. gastrosuis*	1/14	7.1

Regarding the *H. pylori* PCR results, 2/14 samples amplified *H. pylori* DNA with homologies of 100% (Additional file 2: Table S2) using the *ureAB* gene primers but no amplification was achieved when using the *glmM* gene primers. Therefore, these cases were reclassified as *H. pylori*-like.

Positive results for *F. gastrosuis* were obtained in 6 out of the 14 wild boars (42.9%), although it was mainly found in association with helicobacters (4/11 or 36.4%) rather than alone (2/11 or 18.2%). Indeed, in 6/14 of the positive sampled stomachs (42.9.0%), only one species was identified while mixed infections with two or more distinct bacteria were detected in 5/14 (35.7%) (Table 7). The most frequent bacterial combination was *H. salomonis* + *F. gastrosuis* (2/14 or 14.3%) (Table 7).

Bacteria were differently distributed throughout the different regions of wild boars’ stomach (Table 8).

**Table 8:** **Specific** ***Helicobacter*** **spp. and** ***F. gastrosuis*** **detected through PCR in the different regions of wild boars’ stomach**

	Pars oesophagea		*r*	*p*	Oxyntic mucosa		*r*	*p*	Antral mucosa		*r*	*p*
Number of samples	*N* = 14				*N* = 14				*N* = 14			
	*n*/*N*	%			*n*/*N*	%			*n*/*N*	%		
Single bacteria												
*H. pylori-*like	0/14	0.0			1/14	7.1			0/14	0.0		
*H. suis*	0/14	0.0			1/14	7.1			0/14	0.0		
*H. bizzozeronii*	1/14	7.1			1/14	7.1			0/14	0.0		
*H. salomonis*	2/14	14.3			0/14	0.0			3/14	21.4		
*F. gastrosuis*	2/14	14.3			4/14	28.6			5/14	28.5		
Multiple bacteria												
*H. pylori*-like + *F. gastrosuis*	0/14	0.0	–	–	1/14	7.1	–	–	0/14	0.0	–	–
*H. bizzozeronii* + *F. gastrosuis*	1/14	7.1	0.22	0.55	1/14	7.1	–	–	0/14	0.0	–	–
*H. bizzozeronii* + *H. salomonis* + *F. gastrosuis*	0	0	–	–	0	0.0	–	–	1/14	7.1	0.67	0.04

*r*: Pearson correlation coefficient.

*p*: *p*-value ≤ 0.05 was considered to be significant.

Regarding the identification of DNA of only one bacteria per sample site, the *pars oesophagea* samples presented 2/14 (14.3%) DNA for *F. gastrosuis*, 2/14 (14.3%) DNA for *H. salomonis* and 1/14 (7.1%) DNA for *H. bizzozeronii*. The oxyntic mucosa had 2/14 (13.3%) of the samples with *F. gastrosuis* DNA, 1/14 (7.1%) with *H. pylori*-like DNA, 1/14 (7.1%) with *H. suis* DNA and 1/14 (7.1%) with *H. bizzozeronii* DNA. The antral mucosa had 4/14 (28.5%) of the samples with *F. gastrosuis* DNA followed by *H. salomonis* DNA in 3/14 (21.4%) of the samples (Table 8).

Regarding the antral mucosa, there was a statistically significant high degree of correlation between the presence of *H. salomonis* and *H. bizzozeronii* (*r* = 0.67, *p* ≤ 0.05) (Table 8).

Concerning the correlation between the presence of *Helicobacter* spp., *F. gastrosuis* DNA and different histological findings, there was a highly statistically significant correlation between the presence of *H. bizzozeronii* and hyperplasia in the *pars oesophagea* (Table 9).

**Table 9:** **Correlation between the PCR-positive results obtained in the different stomach regions and the gastric histological findings in wild boars**

		Erosion			Ulceration			Hyperplasia			Fibrosis		
	n/N	4/10	*r*	*p*	0/10	*r*	*p*	2/10	*r*	*p*	0/10	*r*	*p*
Pars oesophagea													
*H. pylori*-like	0/10	0	–	–	0	–	–	0	–	–	0	–	–
*H. suis*	0/10	0	–	–	0	–	–	0	–	–	0	–	–
*H. bizzozeronii*	1/10	0	–	–	0	–	–	2/2	0.94	< 0.01	0	–	–
*H. salomonis*	0/10	0	–	–	0	–	–	0	–	–	0	–	–
*F. gastrosuis*	4/10	2/4	0.36	0.31	0	–	–	1/2	0.03	0.93	0	–	–

	n/N	0/10	*r*	*p*	0/10	*r*	*p*	1/10	*r*	*p*	0	*r*	*p*
Oxyntic mucosa													
*H. pylori*-like	2/10	0	–	–	0	–	–	0	–	–	0	–	–
*H. suis*	1/10	0	–	–	0	–	–	0	–	–	0	–	–
*H. bizzozeronii*	0/10	0	–	–	0	–	–	0	–	–	0	–	–
*H. salomonis*	0/10	0	–	–	0	–	–	0	–	–	0	–	–
*F. gastrosuis*	5/10	0	–	–	0	–	–	0	–	–	0	–	–

	n/N	0/10	*r*	*p*	0/10	*r*	*p*	0/10	*r*	*p*	0	*r*	*p*
Antral mucosa													
*H. pylori*-like	0/10	0	–	–	0	–	–	0	–	–	0	–	–
*H. suis*	0/10	0	–	–	0	–	–	0	–	–	0	–	–
*H. bizzozeronii*	2/10	0	–	–	0	–	–	0	–	–		–	–
*H. salomonis*	5/10	0	–	–	0	–	–	0	–	–	0	–	–
*F. gastrosuis*	6/10	0	–	–	0	–	–	0	–	–	0	–	–

*r*: Pearson correlation coefficient.

*p*: *p*-value ≤ 0.05 was considered to be significant.

## Discussion

It is known that the presence of helicobacters may be associated with gastric disease in humans and animals. *F. gastrosuis* has been described to possibly play a role in gastric ulceration in pigs [17, 18].

This study reports a high occurrence of gastritis in pigs and wild boars, 95.8% and 100% respectively, as was previously described by Hessing et al., Robertson et al., De Witte et al. [18, 32, 33].

Regarding the pig samples that presented gastritis: 51.9% of the *pars oesophagea* tissues were positive for one or more *Helicobacter* spp. and *F. gastrosuis* and, specifically 23.1% were positive for a *H. pylori*-like species and 11.5% for a *H. pylori*-like species in combination with *F. gastrosuis* (Additional file 4). In the oxyntic mucosa samples, 50.0% were positive for *Helicobacter* spp. and *F. gastrosuis* but, in contrast, the species most often detected was *H. suis*. In addition, positive cases for *F. gastrosuis* were always accompanied by a *Helicobacter* species (Additional file 4). Among the porcine antral gastritis cases, 57.6% were positive for *Helicobacter* spp. and *F. gastrosuis,* while 36.4% were *H. suis* positive, 7.6% *F. gastrosuis* positive and 6.1% *F. gastrosuis* plus *H. suis* positive (Additional file 4).

In both pigs and wild boars, *F. gastrosuis* was detected in association with *Helicobacter* species which corroborates their potential synergy to induce gastric pathology (Additional files 4 and 5).

Particularly in pigs, *F. gastrosuis* may have a synergetic role with *H. suis* in gastric ulceration as described by De Witte et al. [18]. De Bruyne et al. described a correlation between *H. suis* infection and the development of gastritis [14]. *H. suis* can affect the acid gastric secretion by altering the number and/or function of parietal D and G cells, as well as interfere with the sonic hedgehog (Shh) signaling pathway that regulates the gastric acid secretion and is involved in the gastric organogenesis, glandular differentiation and gastric homeostasis. This may lead to gastroesophageal ulceration as well as affect the gastric microbiota since the presence of *H. suis* alters the gastric environment which may promote the proliferation of other microorganisms such as *F. gastrosuis* in the non-glandular zone leading to gastritis and ulceration [10, 11, 34].

In pig samples, gastric ulceration and erosion were higher in the antral mucosa samples positive for *H. suis* and *F. gastrosuis* and a statistically significant correlation for the presence of *F. gastrosuis* with ulceration (*r* = 0.31, *p* ≤ 0.05), and *H. pylori*-like with erosion (*r* = 0.27, *p* ≤ 0.05) was demonstrated. The presence of *H. suis* and *F. gastrosuis* in pigs was previously reported and it was hypothesized that bacterial interaction can lead to gastric lesions and ulceration of the *pars oesopahea* [10, 18].

Compared to pigs, wild boars had a lower percentage of a *H. pylori*-like species and *H. suis* in the analyzed samples. However, both had close percentages of samples positive for *F. gastrosuis* (35.2% in pigs vs 42.9% in wild boars). Previous studies have reported the presence of *Helicobacter* spp. in wild boars [26, 35, 36], but this remains the first description of *F. gastrosuis* DNA detection and its possible relation with gastric erosion and ulceration in this species, as it has been described in pigs [10, 17, 18].

DNA of zoonotic *Helicobacter* spp. were detected in the pigs’ and wild boars’ stomach samples screened in this study, so the close contact between wildlife, domestic animals, and humans should be a concern for the transmission of bacteria with zoonotic potential that raises awareness in a One health perspective [4, 5, 19, 21, 24–26].

In the present study, DNA from *H. felis*, *H. bizzozeronii* and *H. salomonis*, which naturally colonize the stomach of dogs and cats, was detected in the stomach of pigs and/or wild boars. It is not clear whether these species are really able to colonize the stomach of these animals or whether our results are due to, for instance, environmental contamination.

Two PCR assays were used for detection of *H. pylori* DNA, one targeting the *ureAB* gene and the other one targeting the *glmM* gene. Although a number of studies have been published diagnosing *H. pylori* infection based on a *ureAB* gene PCR only [19, 37, 38], some reports indeed claim that *H. pylori* identification should include these two different target genes amplification [39–41]. Lu et al. compared five PCR methods for detection of *H. pylori* DNA of 24 culture-positive samples obtained from 50 human gastric samples, including PCRs targeting the *ureA* and *glmM* gene. These authors considered the *glmM* gene PCR to be the most appropriate method for detection of *H. pylori* organisms in clinical samples [42]. After analyzing 290 human gastric samples, Elnosh et al*.* concluded that a *glmM* gene based PCR showed higher sensitivity and specificity than a *ureA* gene based PCR [43]. In another investigation, only 10 out of the 50 human gastric samples analyzed turned out positive using the *glmM* gene whereas 25 were PCR-positive using the *ureA* gene [44].

It is remarkable that more than 50% of the pigs were positive in the *ureAB* PCR, while none of the samples were positive in the *glmM* PCR. Sequencing of the amplicons obtained in the *ureAB* PCR, revealed high homologies with *H. pylori ureAB* ranging from 96.3 to 100%. Natural colonization of the stomach of pigs with *H. pylori* has not been described before. We hypothesize that a *H. pylori-*like species, with a similar *ureAB* gene as *H. pylori*, was detected*.* This organism seems to preferably colonize the proximal, non-glandular part of the stomach whereas *H. suis* mainly colonizes the distal part. In any case, our results should be confirmed by isolation and identification of this *H. pylori-*like species from the stomach of pigs.

Krakowka et al. described a *Helicobacter* species present in the stomach of pigs, which had a curve-shaped morphology similar to that of *H. pylori* [45]*.* Unfortunately, no taxonomic studies or genome sequences have been published for this *Helicobacter* species and its exact identity is unclear [10]. Comparison with our results is therefore not possible.

This study reports the presence of a *H. pylori-*like species, gastric NHPH, and *F. gastrosuis* DNA in the stomach of pigs and wild boars and the putative gastric histopathological alterations associated. The results suggest that pigs and wild boars may act as reservoirs for these bacteria.

Further research should be carried out, including studies with a larger sampled population of wild boars, to assess the real prevalence of these bacteria in this animal species and to better understand their possible role in the development of gastric pathology.

## Supplementary Information

**Additional file 1.** Primer sequences used for detection of *Helicobacter* spp. and *F. gastrosuis* and thermocycling conditions.

**Additional file 2.** Nonparametric Kruskal–Wallis, Chi Square test applied to the three gastric zones comparing the different variables in pigs.

**Additional file 3.** BLAST identity percentage interval of the different *Helicobacter* spp. and *F. gastrosuis* sequences obtained.

**Additional file 4.** Number of *Helicobacter* spp. and *F. gastrosuis* DNA positive samples associated with gastritis score per pig gastric zone.

**Additional file 5.** Number of *Helicobacter* spp. and *F. gastrosuis* DNA positive samples associated with gastritis score per wild boar’s gastric zone.
