# Supplementary material for: Molecular detection of Helicobacter spp. and Fusobacterium gastrosuis in pigs and wild boars and its association with gastric histopathological alterations
Source: Vet Res. 2022 Oct 8;53:78. doi: 10.1186/s13567-022-01101-5 (PMC9548099; doi:10.1186/s13567-022-01101-5)
Supplement: Supplementary file 1 — Additional file 1. Primer sequences used for detection of Helicobacter spp. and F. gastrosuis and thermocycling conditions. [file 13567_2022_1101_MOESM1_ESM.docx]

**Additional file 1 Primer sequences used for detection of *Helicobacter* spp. and *F. gastrosuis* and thermocycling conditions**

| ***Helicobacter* species** | **Primer** | **Sequence** | **Target gene** | **Amplicon size** | **Thermal cycle conditions** | | | ***Helicobacter* strain used as positive control** |
| --- | --- | --- | --- | --- | --- | --- | --- | --- |
|  |  |  |  |  | **Nr. Cycles** | **Temp. (ºC)** | **Time** |  |
| *H. suis* | BFHsuis_F1 | AAA ACA MAg gCg ATC gCC CTg TA | *ureA* | 150bp | 40 | 95  60  72 | 20 sec  30 sec  30 sec | HS1 |
|  | BFHsuis_R1 | TTT CTT CgC CAg gTT CAA AgC g | *ureA* |  |  |  |  |  |
| *H. heilmannii* | Hh-IceA-FWQ | gTT TCC AAC CAA AAg ACT CA | *iceA* | 135 bp | 30 | 94  55  72 | 30 sec  30 sec  30 sec | ASB1.4 |
|  | Hh-IceA-RVQ | ATT gCC TAg Agg TTg TgT Tg | *iceA* |  |  |  |  |  |
| *H. ailurogastricus* | Ha-LpsA-FWQ | CTT gAg TAC ggC gAT gTC AAT | *lpsA* | 136 bp | 30 | 94  55  72 | 30 sec  30 sec  30 sec | ASB7.1 |
|  | Ha-LpsA-RVQ | ggg gAA AAA TgT gCT TgA AgT | *lpsA* |  |  |  |  |  |
| *H. salomonis* | Hsal_FQ_PAR | CTC TTA TgA gTT ggA CTT ggT gCT CAC CAA T | *ureAB* | 91 bp | 45 | 94  61  72 | 30 sec  30 sec  1 min | R1051 |
|  | Hsal_RQ_PAR | TTT gCC ATC TTT AAT TCC AAT gTC ggC | *ureAB* |  |  |  |  |  |
| *H. felis* | BFHfel_F2 | gCT ggT ggC ATC gAT ACg CAT | *ureAB* | 154 bp | 45 | 94  60  72 | 30 sec  30 sec  1 min | CS1 |
|  | BFHfel_R2 | TTT TTA gAT TAg CgC gTC Cgg gA | *ureAB* |  |  |  |  |  |
| *H. bizzozeronii* | Hbizz_FQ_PAR | CCA ACA AAT CCC CAC AgC ATT TgC CAg | *ureAB* | 91 bp | 45 | 94  58  72 | 1 min  1 min  1 min | R1053 |
|  | Hbizz_RQ_PAR | AgT CCC ATC AgC Wgg WCC TgT TCC CCC AC | *ureAB* |  |  |  |  |  |
| *H. pylori* | BFHpyl_F1 | AAA gAg CgT ggT TTT CAT ggC g | *ureAB* | 217 bp | 45 | 94  59  72 | 30 sec  30 sec  1 min | 26695 |
|  | BFHpyl_R1 | ggg TTT TAC CgC CAC CgA ATT TAA | *ureAB* |  |  |  |  |  |
|  | Hpy3F | TTATCGGTAAAGACACCAGAAA | *glmM* | 144 | 45 | 94  54  72 | 30 sec  30 sec  30 sec | SS1 |
|  | Hpy3R | ATCACAGCGCATGTCTTC | *glmM* |  |  |  |  |  |
| *F. gastrosuis* | GB_F | GCA GCT CAA AGA GCA AGA GAA GCA | *gyrB* | 158bp | 35 | 95  63  72 | 20 sec  30 sec  30 sec | CDW1T |
|  | GB2_R | CTT CCC TGC TTT GCA GAA CCT CC | *gyrB* | 158bp |  |  |  |  |
