# Supplementary material for: Molecular detection of Helicobacter spp. and Fusobacterium gastrosuis in pigs and wild boars and its association with gastric histopathological alterations
Source: Vet Res. 2022 Oct 8;53:78. doi: 10.1186/s13567-022-01101-5 (PMC9548099; doi:10.1186/s13567-022-01101-5)
Supplement: Supplementary file 2 — Additional file 2. Nonparametric Kruskal–Wallis, Chi Square test applied to the three gastric zones comparing the different variables in pigs. [file 13567_2022_1101_MOESM2_ESM.docx]

**Additional file 2 Nonparametric Kruskal-Wallis, Chi Square test applied to the three gastric zones comparing the different variables in pigs.**

|  | **finsco** | **fib** | **eros** | **ulc** | **hyperp** | **hpyl** | **hsuis** | **hfelis** | **hsal** | **fuso** |
| --- | --- | --- | --- | --- | --- | --- | --- | --- | --- | --- |
| **Kruskal-Wallis Test** | | | | | | | | |  |  |
| Chi-Square | 9.12 | 69.18 | 6.24 | 33.39 | 77.95 | 36.44 | 17.05 | 3.58 | 3.75 | 6.385 |
| DF | 2 | 2 | 2 | 2 | 2 | 2 | 2 | 2 | 2 | 2 |
| Pr > Chi-Square | **0.0105** | **<.0001** | **0.0442** | **<.0001** | **<.0001** | **<.0001** | **0.0002** | 0.1666 | 0.1533 | **0.0411** |

finsco – final score of gastritis; neut – neutrophils; eosin – eosinophils; fib – fibrosis; eros – erosion; hpyl – H*. pylori*; hsuis *– H. suis*; hfelis – *H. felis*; hsal – *H. salomonis;* fuso – *F. gastrosuis*; - *p* < 0.05 – statistically significant differences between gastric zones (*pars esopahea*, *antrum* and oxyntic mucosa)
