# Supplementary material for: Molecular detection of Helicobacter spp. and Fusobacterium gastrosuis in pigs and wild boars and its association with gastric histopathological alterations
Source: Vet Res. 2022 Oct 8;53:78. doi: 10.1186/s13567-022-01101-5 (PMC9548099; doi:10.1186/s13567-022-01101-5)
Supplement: Supplementary file 3 — Additional file 3. BLAST identity percentage interval of the different Helicobacter spp. and F. gastrosuis sequences obtained. [file 13567_2022_1101_MOESM3_ESM.docx]

**Additional file 3 BLAST identity percentage interval of the different *Helicobacter* spp. and *F. gastrosuis* sequences obtained.**

| **Animals** | **Bacteria** | **Identity** | **Reference strain Accession Number** |
| --- | --- | --- | --- |
| Pigs | *H. pylori* | 96.3-100% | CP048599 |
|  |  | 99.2-100% | CP024946 |
|  |  | 99.3-100% | CP032027 |
|  |  | 98.6% | CP048600 |
|  |  | 100% | CP011330 |
|  |  | 98.1-100% | CP032037 |
|  | *H. suis* | 95.6-100% | AP023036 |
|  |  | 96.8-100% | AP023046 |
|  | *H. felis* | 95.9% | JQ736360 |
|  |  | 97.1% | FQ670179 |
|  | *H. salomonis* | 100% | AJ130882 |
|  | *F. gastrosuis* | 96.0-100% | LN906798 |
| Wild Boars | *H. bizzozeronii* | 95.3-99.2% | FR871757 |
|  | *H. felis* | 99.1% | FQ670179 |
|  | *H. suis* | 98.9% | AP023046 |
|  | *H. pylori* | 100% | AF507994 |
|  |  | 100% | AY368264 |
|  | *H. salomonis* | 96.4% | AF508005 |
|  |  | 100% | AY368266 |
|  | *F. gastrosuis* | 97.0-100% | LN906798 |
