# Supplementary material for: Molecular detection of Helicobacter spp. and Fusobacterium gastrosuis in pigs and wild boars and its association with gastric histopathological alterations
Source: Vet Res. 2022 Oct 8;53:78. doi: 10.1186/s13567-022-01101-5 (PMC9548099; doi:10.1186/s13567-022-01101-5)
Supplement: Supplementary file 4 — Additional file 4. Number of Helicobacter spp. and F. gastrosuis DNA positive samples associated with gastritis score per pig gastric zone. [file 13567_2022_1101_MOESM4_ESM.docx]

|  | *Pars oesophagea* | | | | | Oxyntic Mucosa | | | | | Antral mucosa | | | | |
| --- | --- | --- | --- | --- | --- | --- | --- | --- | --- | --- | --- | --- | --- | --- | --- |
|  | Normal | Mild  gastritis | Moderate  gastritis | Severe  gastritis | Total | Normal | Mild  gastritis | Moderate gastritis | Severe gastritis | Total | Normal | Mild  gastritis | Moderate  gastritis | Severe  gastritis | Total |
|  | *n* = 5 | *n* = 16 | *n* = 9 | *n* = 27 | *n* = 57 | *n* = 9 | *n* = 28 | *n* = 13 | *n* = 19 | *n* = 69 | *n* = 0 | *n* = 2 | *n* = 3 | *n* = 61 | *n* = 66 |
| *H. pylori*-like | 1 | 6 | 0 | 6 | 13 | 1 | 3 | 2 | 1 | 7 | 0 | 0 | 0 | 1 | 1 |
| *H. suis* | 0 | 0 | 0 | 0 | 0 | 3 | 10 | 2 | 5 | 20 | 0 | 0 | 2 | 22 | 24 |
| *H. felis* | 0 | 0 | 0 | 0 | 0 | 0 | 1 | 0 | 0 | 1 | 0 | 0 | 0 | 0 | 0 |
| *H. salomonis* | 0 | 0 | 0 | 0 | 0 | 0 | 0 | 0 | 0 | 0 | 0 | 0 | 0 | 2 | 2 |
| *F. gastrosuis* | 0 | 3 | 0 | 0 | 3 | 0 | 0 | 0 | 0 | 0 | 0 | 0 | 1 | 4 | 5 |
| *H. pylori*-like *+ H.suis* | 0 | 1 | 1 | 3 | 5 | 0 | 0 | 1 | 0 | 1 | 0 | 0 | 0 | 1 | 1 |
| *H.suis + H. felis* | 0 | 0 | 0 | 0 | 0 | 0 | 0 | 0 | 1 | 1 | 0 | 0 | 0 | 0 | 0 |
| *H. pylori*-like *+ F. gastrosuis* | 2 | 2 | 0 | 4 | 8 | 0 | 0 | 0 | 1 | 1 | 0 | 0 | 0 | 1 | 1 |
| *H. suis + F. gastrosuis* | 0 | 0 | 0 | 0 | 0 | 0 | 1 | 1 | 0 | 2 | 0 | 0 | 0 | 4 | 4 |
| *H. pylori*-like *+ H.suis + F. gastrosuis* | 0 | 0 | 1 | 0 | 1 | 0 | 1 | 0 | 0 | 1 | 0 | 0 | 0 | 0 | 0 |

**Additional file 4 Number of *Helicobacter* spp. and *F. gastrosuis* DNA positive samples associated with gastritis score per pig gastric zone.**
