# Supplementary material for: Molecular detection of Helicobacter spp. and Fusobacterium gastrosuis in pigs and wild boars and its association with gastric histopathological alterations
Source: Vet Res. 2022 Oct 8;53:78. doi: 10.1186/s13567-022-01101-5 (PMC9548099; doi:10.1186/s13567-022-01101-5)
Supplement: Supplementary file 5 — Additional file 5. Number of Helicobacter spp. and F. gastrosuis DNA positive samples associated with gastritis score per wild boar’s gastric zone. [file 13567_2022_1101_MOESM5_ESM.docx]

**Additional file 5 Number of *Helicobacter* spp. and *F. gastrosuis* DNA positive samples associated with gastritis score per wild boar’s gastric zone**

|  | *Pars oesophagea* | | | | | Oxyntic mucosa | | | | | Antral mucosa | | | | |
| --- | --- | --- | --- | --- | --- | --- | --- | --- | --- | --- | --- | --- | --- | --- | --- |
|  | Normal | Mild  gastritis | Moderate  gastritis | Severe  gastritis | Total | Normal | Mild  gastritis | Moderate  gastritis | Severe gastritis | Total | Normal | Mild  gastritis | Moderate  Gastritis | Severe  gastritis | Total |
|  | *n* = 2 | *n* = 4 | *n* = 2 | *n* = 2 | *n* = 10 | *n* = 6 | *n* = 1 | *n* = 2 | *n* = 1 | *n* = 10 | *n* = 1 | *n* = 1 | *n* = 1 | *n* = 7 | *n* = 10 |
| *H. pylori*-like | 0 | 0 | 0 | 0 | 0 | 0 | 0 | 0 | 0 | 0 | 0 | 0 | 0 | 0 | 0 |
| *H. suis* | 0 | 0 | 0 | 0 | 0 | 0 | 0 | 0 | 0 | 0 | 0 | 0 | 0 | 0 | 0 |
| *H. bizzozeronii* | 0 | 0 | 0 | 1 | 1 | 1 | 0 | 0 | 0 | 1 | 0 | 0 | 0 | 0 | 0 |
| *H. salomonis* | 0 | 0 | 1 | 0 | 1 | 0 | 0 | 0 | 0 | 0 | 0 | 0 | 0 | 1 | 1 |
| *F. gastrosuis* | 0 | 1 | 0 | 1 | 2 | 1 | 1 | 0 | 0 | 1 | 1 | 0 | 0 | 2 | 3 |
| *F.gastrosuis + H. bizzozeronii* | 0 | 1 | 0 | 0 | 1 | 0 | 0 | 1 | 0 | 1 | 0 | 0 | 0 | 0 | 0 |
| *H.bizzozeronii + H. salomonis + F. gastrosuis* | 0 | 0 | 0 | 0 | 0 | 0 | 0 | 0 | 0 | 0 | 0 | 0 | 0 | 1 | 1 |
